# Circulating tumor cells and circulating tumor DNA in breast cancer diagnosis and monitoring

**DOI:** 10.32604/or.2023.028406

**Published:** 2023-07-21

**Authors:** EFFAT ALEMZADEH, LEILA ALLAHQOLI, HAMIDEH DEHGHAN, AFROOZ MAZIDIMORADI, ALIREZA GHASEMPOUR, HAMID SALEHINIYA

**Affiliations:** 1 Infectious Diseases Research Center, Birjand University of Medical Sciences, Birjand, 9717853577, Iran; 2 Midwifery Department, Ministry of Health and Medical Education, Tehran, 9413933336, Iran; 3 Student Research Committee, Birjand University of Medical Sciences, Birjand, 9717853577, Iran; 4 Department of Health Assistant, Shiraz University of Medical Sciences, Shiraz, 7134814336, Iran; 5 Social Determinants of Health Research Center, Birjand University of Medical Sciences, Birjand, 32048321, Iran

**Keywords:** Breast cancer, Liquid biopsy, Circulating tumor cells, Circulating tumor DNA

## Abstract

Liquid biopsy, including both circulating tumor cells and circulating tumor DNA, is becoming more popular as a diagnostic tool in the clinical management of breast cancer. Elevated concentrations of these biomarkers during cancer treatment may be used as markers for cancer progression as well as to understand the mechanisms underlying metastasis and treatment resistance. Thus, these circulating markers serve as tools for cancer assessing and monitoring through a simple, non-invasive blood draw. However, despite several study results currently noting a potential clinical impact of ctDNA mutation tracking, the method is not used clinically in cancer diagnosis among patients and more studies are required to confirm it. This review focuses on understanding circulating tumor biomarkers, especially in breast cancer.

## Introduction

Breast cancer (BC) is the most prevalent malignancy affecting women worldwide. The 5-year survival rate in individuals with a localized breast cancer diagnosis and no nodal involvement is 99% compared to 27% for patients with distant metastatic disease [1–3]. Breast cancer is a heterogeneous and complex condition with a wide range of treatment responses, where the incidence of cancer rises with age due to an accumulation of somatic mutations in the mammary glands [4,5]. Despite improvements in the treatment of breast cancer, more than 8.2 million individuals still die every year due to a lack of a reliable method to detect the disease early and to monitor the response to therapy effectively [6,7]. Among all new cases, 6%–7% are diagnosed with de-novo metastatic disease, and about 30% of patients initially identified in earlier stages eventually relapse in distant sites. As a result, it is always a challenge for researchers to consider methods to help with cancer detection and monitoring. In addition, the techniques used today for the early detection of breast cancer have remained unchanged for more than 20 years and can be invasive to the patient’s physical health during the diagnosis process. For example, in radiology, ultrasound detection, and MRI scans, solid biopsies are commonly used methods for cancer detection which are limited by poor sensitivity, overdiagnosis, false-positive rates, ineffective detection of minimal residual disease, and incapable of monitoring dynamic changes [8,9].

Thus, a breast cancer prognosis worsens with the absence of young women’s early screening programs and efficient diagnostic tools in general [10]. These drawbacks emphasize how critical it is to create new tools and methods for the early detection and management of breast cancer. Liquid biopsy is a new minimally invasive technique for the early detection and risk management of breast cancer. Since liquid biopsy is a technique for examining nonsolid biological tissues, hence it has good potential to overcome the drawbacks of conventional approaches [11,12].

The purpose of this review is to summarize technologies for *Circulating tumor cells (CTCs)* and *circulating tumor DNA (ctDNA)* detection as well as their clinical applications as complementary tools to improve the outcome of patients with breast cancer.

## Liquid Biopsy Markers

*CTCs* and *ctDNA* are new, noninvasive, and multifunctional biomarkers used in the liquid biopsy which enable early diagnosis, precise prognosis, therapeutic target selection, spatiotemporal monitoring of metastasis, and monitoring of therapeutic response as well as resistance. Different tumor types at different stages release *CTCs* and *ctDNA*, which could provide complementary information for clinical decisions [11].

### CTCs

Since the 1860s, when tumor cells were first discovered in patients’ peripheral blood, there have been tremendous advancements in the ability to isolate *CTCs* from a diverse population of blood cells [12]. *CTCs* are released from primary tumors, travel via the circulatory system, and are responsible for the growth of metastatic (or secondary) malignancies at distant locations throughout the body [13]. Their percentage in the blood is very low, with only around one *CTC* being identified for every million leukocytes [14]. According to morphological studies, *CTCs* have different shapes based on the stage and/or kind of the tumor C. Furthermore, compared to their isolated *CTC* counterparts, aggregated *CTCs* have been shown to spread to further locations in the body after affixing to cells such as fibroblasts, platelets, etc. Thus, these cellular aggregates are shielded from oxidative damage and can evade detection by the immune system of the host organism, which can contribute to the development of metastases [15,16].

*CTCs* have become increasingly important in the detection of cancers due to their ease of collection and ability to provide real-time information about the tumor’s state without the need for invasive tissue biopsies. Unlike typical blood indicators, *CTC* levels have been shown to change in a more dynamic manner, closely tracking changes in the tumor status with greater precision [17]. Additionally, *CTC* counts have been shown to be a more accurate predictor of treatment response, with lower *CTC* counts being associated with improved overall survival in a large cohort of breast cancer patients [18]. Additionally, *CTCs* have demonstrated encouraging outcomes in the early identification of numerous cancer forms, including lung cancer, though only in a small subset of individuals with chronic obstructive pulmonary disease [19].

### ctDNA

A highly effective diagnostic method based on a patient’s genetic and epigenetic makeup has been made possible by *ctDNA* in precision medicine [20]. *ctDNA* is highly heterogeneous both in size and composition, and can be detected in different body fluids [21]. The mechanism though which *cfDNA* is released by cells is yet to be fully clarified. Electrophoresis assays demonstrated that most fragments range between 180 and 200 base pairs (bp) and are often associated with histone proteins that form the nucleosome, suggesting that apoptotic cells could be one of the most important source of *cfDNA* [22,23]. These observations were strongly related to a rapid increase in circulating nucleosomes during anticancer treatments and by a rapid decline at disease progression, supporting the idea that the quantification of nucleosome bodies can represent an efficient index of responsiveness to the therapy [24].

In cancer patients, ctDNA is found in a variable but usually very low percentage (0.01%–1.0%) of the total *cfDNA*, which is usually less than 1 ng/μL, and varies depending on the stage, location, or vascularization of the tumor [25]. The global *cfDNA* can be easily quantified and is known to rise in breast cancer patients compared to healthy subjects [26]. The concentration of circulating cell-free *DNA* among healthy individuals and those with various types of cancer was measured at 13 ± 3 ng/mL and 180 ± 38 ng/mL, respectively [27].

*ctDNA* detection was significantly associated with the molecular subtypes, with the Basal-like and the Luminal-A subtypes showing the highest (86%) and lowest (0%) *ctDNA* detection rates, respectively [28]. Elevated levels of circulating tumor cells have been associated with a worse prognosis [29,30]. In contrast to early, non-metastatic breast cancer, *ctDNA* is detectable in the majority of metastatic breast cancers. Zhou et al. reported that 85.71% of stage IV/M1 patients carried tumor-derived mutations in blood, compared to only 57.81% of stage I–III/M0 patients [31]. In another study, Catarino et al. reported elevated levels of circulating *DNA* in breast cancer patients compared to control individuals (105.2 *vs*. 77.06 ng/mL, *p* < 0.001). They also found statistically significant differences in circulating *DNA* amounts in patients before and after breast surgery (105.2 *vs*. 59.0 ng/mL, *p* = 0.001) [32].

*ctDNA* is also related to tumor volume in breast, ovarian, lung, colorectal, and stomach cancers, which results in a reduced overall survival time [33]. Contradictory findings from various studies suggest that concentration is not related to general or advancement-free existence [34]. The limitations of *ctDNA* for diagnostic purposes can be inferred from that research, though ctDNA can still be employed to track tumor growth. Clinical uses for *ctDNA* include disease monitoring and diagnosis. Through the use of *ctDNA*, certain studies have been able to locate mutations in a patient’s tumor. For instance, a *PIK3CA* mutation provided a 95% accurate diagnosis of breast cancer [35]. As a result, *ctDNA* may be utilized to detect interesting mutations and genetic heterogeneity. *CtDNA* can also be used to monitor the effects of treatment by looking for mutation-driven resistance [36]. Endocrine treatment resistance to the *ESR1* mutation is a frequent feature in patients with metastatic breast cancer [37]. Early diagnosis of this type of mutation and treatment prior to clinical progression are both conceivable utilizing *ctDNA* [38].

## CTC

### Technologies for CTC detection and characterization

**Figure 1:**
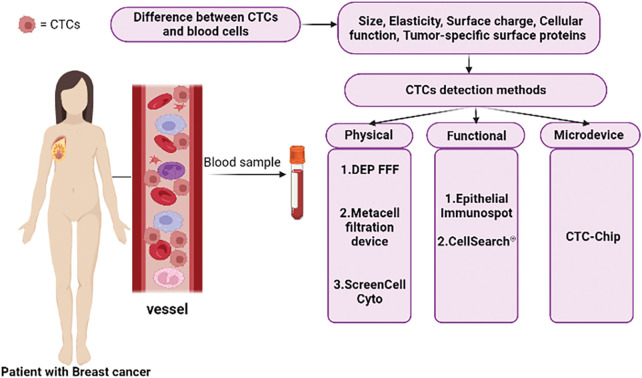
Summary of CTCs detection methods in patient blood samples and the basis of these methods.

*CTCs* are uncommon and infrequent neoplastic cells that should be detected using a high-level detection platform, the proper tools, and methodologies because of their low peripheral blood concentration (almost 1 cell per 105 to 107 mononuclear cells) [39,40]. Despite the availability of numerous techniques and technologies for identifying CTCs based on their physical (such as size, elasticity, and surface charge) and biological (such as cellular function) features as well as the expression of tumor-specific surface protein characteristics (Fig. 1), the Food and Drug Administration (*FDA*) has only approved the CellSearch® system as a platform for counting CTCs thus far [41,42].

The CellSearch® was designed to enable fluorescent labelling, immunomagnetic enrichment, and detection of *CTCs*. This approach utilizes ferrofluid nanoparticles coated with *EpCAM*-targeting antibodies in a *CTC*-enrichment stage, allowing for efficient capture of *CTCs* from blood samples [41,43]. This strategy can be used to monitor patients after chemotherapy or surgery, as well as to treat breast cancer and other cancers [39]. However, this approach is time-consuming, requires expensive tools, and involves antibody staining, which would hamper the wide application of this technology [40]. CellSearch®’s enrichment approach results in cell loss, which affects the system’s sensitivity despite the technology’s proven clinical validity. Additionally, it only selects *CTCs* that express *EpCAM*, missing other *CTC* phenotypes such as mesenchymal and stem cell-like tumor cells that express *EpCAM* at low or zero levels [41].

In addition to Cell Search as an immunobead assay, there are other methods and approaches for detecting *CTCs*, including physical property-based assays (such as *Dielectrophoretic field-flow fractionation (DEP-FFF)*, the *Metacell filtration device, ScreenCell Cyto*, etc.), functional assays (such as the *Epithelial Immunospot (EPISPOT)* assay, and etc.), *microdevices*, as well as microfluidic platforms (such as the *CTC-Chip*, which consists of *microposts* coated with *anti-EpCAM* antibodies, and etc.) (Fig. 1) [42].

Kong et al. [44] used the Drop Cell as a single-cell isolation platform for *CTC* isolation at single-cell resolution. In order to assess the genetic heterogeneity of *ctDNA* and *CTCs* in patients with lung and breast cancer, they actually constructed a standardized sample processing methodology which allowed for the concurrent separation of *CTC* and *ctDNA* followed by targeted amplicon sequencing. To isolate *CTCs* at single-cell resolution, they used *CD45*-antibody negative selection employing a single-cell capture. The results of this study indicated that *CTCs* and *ctDNA* had a higher degree of concordance with the metastatic tumor than with the primary tumor. As a conclusion, a standardized sample processing and data analysis workflow for the concurrent analysis of *CTCs* and *ctDNA* was successful in separating the heterogeneity of metastatic tumors that were circulating as well as the progressive genomic changes that may guide the selection of an appropriate therapy against evolving tumor clonality [44].

Based on cell size and deformability, Lopes et al. evaluated and contrasted the *RUBYchipTM* (size-based microfluidic device) system’s performance *vs*. that of the Cell Search® system for *CTC* capture. In isolated CTCs, the expression of HER2 was evaluated and compared to tissue biopsy. The study found that, on average, the *RUBYchipTM* was up to ten times more effective at isolating *CTCs* compared to the Cell Search® system. Additionally, a precise assessment of various *CTC* subpopulations, including HER2+ CTCs, was given. The study’s results indicated that liquid biopsy, when used in the clinic with the *RUBYchipTM*, can overcome the limitations of histological testing and determine a patient’s *HER2* status in real-time, allowing for more precise treatment planning as the disease progresses [41].

### Clinical application of CTC

*CTC* detection has been proven in patients with both early-stage and metastatic breast cancer [45,46]. Among the benefits of using *CTC* are ease of collection, non-invasiveness, the possibility of continuous evaluation, as well as analysis of the total tumor burden rather than a limited part of a tumor. Currently, *CTC* is used in breast cancer to gain prognostic information, monitor the effectiveness of treatment and therapeutic interventions, detect the disease stages early, investigate drug sensitivity, and discover new drugs for personalized treatment [47]. However, the practical use of *CTCs* is limited due to their rarity and heterogeneity, as well as problems with initial culture, necessitating the development of easier detection methods compared to the existing ones [48]. It appears that *CTC* count is a criterion for evaluating treatment efficacy, and patients with metastatic breast cancer who had a higher *CTC* count experienced more metastases over time [49]. Also, *CTC* cells appear to be a better model for studying the malignant behavior of breast cancer than existing cell lines. Zhao et al. revealed that the *CTC-3* cell line grows more aggressively *in vitro* and *in vivo* than the commonly used *MCF-7* breast cancer cell line [50].

### In early stage breast cancer

Given the numerous benefits of *CTCs* in the diagnosis and early treatment of breast tumors, the presence of *CTC* clusters in primary breast cancer may be considered an important risk factor for disease progression [47]. Further research is required, however, to address the prognostic potential of *CTC* in the early stages of breast cancer [46].

Kroll et al. discovered the peripheral blood circulation of CTCs in patients with primary non-metastatic breast cancer, shortly after diagnosis and prior to surgical intervention for therapeutic purposes [46].

Using conventional and epithelial-based methods, it is difficult to analyze and identify *CTCs* in the early stages of breast cancer. However, the Reduzzi et al.’s study showed that marker-independent approaches for *CTC* evaluation would improve diagnosis and that CTC is more common in patients with early-stage breast cancer than in patients with metastatic breast cancer [43].

### In metastatic breast cancer

Elevated levels of CTCs have been confirmed as an independent prognostic factor in metastatic breast cancer. Xie et al. discovered that CTC had a prognostic value in patients with metastatic breast cancer. In this study, 38 patients with metastatic breast cancer were enrolled. *CTCs*, collected *in vivo* by the Cell Collector method in Chinese patients with metastatic breast cancer, showed prognostic significance. It seems that the increase of *CTCs* in the blood is a sign of the progression of cancer and the worsening of the disease. This is because, as the number of *CTCs* in the blood increases, so do the genetic heterogeneity of these cells and their interaction with the internal environment of the body [45]. Stefanovic et al. showed that in patients with metastatic breast cancer, CTCs might not only develop their genetic potential but also communicate with their surroundings, including chemokine systems, hemocytes, and extracellular matrix components, to regulate the organ-specific metastases of breast cancer. It appears that the degree of *HER2* status matching between *CTCs* and primary tumors or metastatic sites can reach 77% or 67%, implying that studying *CTC HER2* expression can guide clinical *HER2*-targeted therapy [51]. Deutsch et al. examined the *CTC* status of 264 patients with metastatic breast cancer before and after 4 weeks of a new line of palliative systemic therapy. According to the findings, *HER2*-targeted therapy appears to reduce the overall *CTC* count in patients with metastatic breast cancer [52].

## CtDNA

### Technologies for DNA detection and characterization

**Figure 2:**
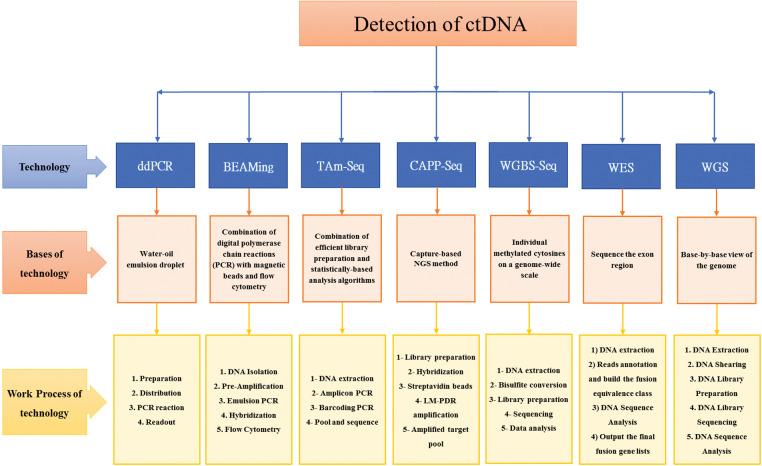
Summary detection of *ctDNA*: Technologies, bases of technologies, and work process of technologies [53–63].

Fig. 2 provides a summary of the technologies, their underlying principles, and the workflow used for the detection of *ctDNA*.

The main techniques employed to evaluate *ctDNA* are: droplet digital polymerase chain reaction (ddPCR), beads, emulsion, amplification, and magnetics (BEAMing), tagged-amplicon deep sequencing (TAm-Seq), cancer personalized profiling by deep sequencing (CAPP-Seq), whole genome bisulfite sequencing (WGBS-Seq), whole exome sequencing (WES), and whole genome sequencing (WGS) [59,64–66].

An important method in translational cancer research is droplet digital polymerase chain reaction (ddPCR), because of its superior sensitivity and precision for genomic *DNA* detection or *RNA* expression [67,68], and could be useful for detecting *HER2* amplification levels [69]. However, it can only be used to evaluate the presence of characterizing sequences [65]. In breast cancer, the ddPCR results had high concordance with FISH and IHC-defined *HER2* status with a sensitivity of 90.9% and a specificity of 100% [69].

BEAMing combines PCR with flow cytometry and is an alternative sensitive approach which provides molecular information about mutations with a frequency of 1 over 10000 [65,70].

Tagged-amplicon deep sequencing (TAm-Seq) enhanced the identification of low frequency mutations in ctDNA [71]. The main features of TAm-Seq include a high sequencing flux, reduced sequencing time plus cost, and the ability to simultaneously sequence millions of DNA molecules, thereby enabling the analysis of the transcripts and genomes of a species in detail [66]. The detection rate of TAm-Seq was reported >2% [53], with sensitivity and specificity of ~100% [71].

CAPP-Seq is a recent NGS-based *ctDNA* analysis method that achieves both an ultralow detection limit and broad patient coverage at a reasonable cost and low *ctDNA* input level, thus allowing the quantitation of ctDNA from early-stage tumors [72–74]. CAPP-Seq identifies alterations in *ctDNA/cfDNA* using large genomic libraries and individual patient sample sequence signatures. It statistically assesses well-characterized tumor alterations with *DNA* oligonucleotides to find patient-specific alterations. It can identify multiple mutations in patients with the same type of cancer and assess tumor heterogeneity. It was previously shown to be capable of identifying tumor burdens prior to medical imaging. It can identify many major mutation types including insertions, deletions, single nucleotide variants, copy variants, and rearrangements, but cannot identify fusions [60,66].

Whole genome bisulfite sequencing (WGBS-Seq) is the gold-standard approach to acquiring comprehensive base-pair resolution and quantitative information at most genomic methylated cytokines, allowing for unbiased genome-wide *DNA* methylation profiling [54]. WGBS is an effective and reliable strategy to identify individually methylated cytosines on a genome-wide scale [75]. Its sensitivity may be lower than other methods, since it includes exomic alterations. It is characterized by low cost and high yield [76].

Whole Genome Sequencing (WGS) and Whole Exome Sequencing (WES) technologies provide novel opportunities for comprehensive profiling of *ctDNA* by detecting genome-wide rearrangements, somatic chromosomal aberrations and Copy Number Variations [64]. These technologies have become increasingly faster, more sensitive, and cost-effective, making their clinical application more feasible. With the ability to sequence at greater depth, WGS and WES offer a promising approach for accurate and sensitive detection of *ctDNA* in clinical settings [77,78].

### Clinical application of CtDNA

Tumor *DNA* in the blood is a result of various mechanisms including apoptosis, necrosis, and circulating tumor cell lysis causing a *DNA* leak into the bloodstream [79]. Various studies have reported a direct correlation between serum levels and tumor burden in breast cancer patients [26,32,80–85], but a diagnostic threshold has not yet been defined [86].

### In early stage breast cancer

Recent advancements in ctDNA technologies have improved sensitivity and selectivity, allowing *ctDNA* to be detected in early-stage disease, including early-stage breast cancer [87]. In early breast cancer, *ctDNA* clearance has been associated with higher rates of complete pathological response after neoadjuvant treatment and with fewer recurrences after radical treatments [88]. Generally, the current clinical application of *ctDNA* for breast cancer involves real-time monitoring of tumor response [89], detecting drug-resistant clones [90,91], assessing dynamic variations in tumor mutational landscape [92], identifying actionable mutations [93], detecting minimal residual disease [28] and screening of early tumor [94,95]. Since there is no wildly accepted baseline level of *ctDNA* for breast cancer diagnosis, variations of *ctDNA* over time do seem to reflect the burden of the disease, determine the prognosis of cancer patients, and help predict therapeutic response [96].

### In metastatic breast cancer

In metastatic disease, *ctDNA* can aid in selecting the optimal sequencing of treatments [88]. Studies have shown that *ctDNA* is a reliable tool for monitoring tumor burden dynamics in patients with metastatic breast cancer undergoing systemic therapy [97]. Compared to traditional biomarkers such as *CA* 15-3 or circulating tumor cells, *ctDNA* levels have a wider dynamic range and exhibit a stronger correlation with changes in tumor burden. Additionally, *ctDNA* analysis provides an early measure of treatment response, with up to 53% of patients showing a response within a few weeks of treatment initiation [85]. Serial analysis of *ctDNA* can also help track clone evolution and predict the development of resistance to therapy, enabling clinical decisions to be made in a timely manner and sparing patients from ineffective treatments. Since *ctDNA* is believed to be shed by all tumor sites, it has the potential to be a useful tool for addressing tumor heterogeneity and therapeutic resistance in both the adjuvant and metastatic settings, guiding therapy decisions more effectively [96].

Liu et al. [98] demonstrated that higher levels of *ctDNA* alterations (levels 3–4) were associated with an increased likelihood of liver metastasis. In addition, a novel *ctDNA*-level *RECIST (ctle-RECIST)* was developed to evaluate treatment response based on *ctDNA* alteration levels and variant allele frequency.

## Conclusion and Future Directions

One of the most pressing challenges in breast cancer treatment is how to better manage patients with early disease. In recent years, research on the applications of liquid biopsies, including *CTCs* and *ctDNA*, has increased dramatically to enhance early diagnosis, detection of recurrence, and personalized treatment. CTCs and *ctDNA* have great potential for determining prognosis, monitoring therapy, and integrating data processing methodologies. Combining *ctDNA* and *CTC* analysis may further improve their prognostic value and clinical utility. As precision and personalized medicine become increasingly important, *CTC* and *ctDNA* monitoring can help detect and subtype the disease and identify patients at high risk of relapse. However, the clinical utility of *ctDNA* mutation tracking needs further investigation before it can become a standard of care for patients with early-stage breast cancer. Standardization of the blood collection process to enhance sample stability, defining *ctDNA* quantification methods, standardizing *ctDNA* isolation, and enhancing the sensitivity of *ctDNA* detection for rare molecular alterations are among the challenges that need to be addressed.

In conclusion, the next generation of liquid biopsy studies will be crucial in establishing the clinical applicability of blood-based genomic profiling. Liquid biopsy techniques offer physicians a relatively inexpensive and non-invasive method for detecting and monitoring early-stage cancers, but it remains to be seen whether treatments based on liquid biopsies will lead to better outcomes.

## Data Availability

The data and material used in the current study are available from the corresponding author upon reasonable request.
